# On the functionalization of benzo[*e*][2,1]thiazine

**DOI:** 10.3762/bjoc.5.42

**Published:** 2009-09-02

**Authors:** Kirill Popov, Tatyana Volovnenko, Julian Volovenko

**Affiliations:** 1 Organic Chemistry Department, Kiev State University, Kiev, 01033, Ukraine

**Keywords:** heterocycles, nucleophilic aromatic substitutions, oxidation, reduction, regioselectivity

## Abstract

The reactions of benzo[*e*][2,1]thiazine-4-chloro-3-carbaldehydes **1** and benzo[*e*][2,1]thiazine-4-chloro-3-carbonitriles **2** with a number of oxidizing and reducing agents are reported. A number of new, highly functionalized benzo[*e*][2,1]thiazine derivatives having potential biological activity were synthesized and described.

## Introduction

Many derivatives of benzothiazine exhibit biological activity that ranges from antipsychotic [1] to anti-inflammatory [2], depending on the substituents present [3–4]. However, medicinal applications are limited as a consequence of side effects [5–6]. Much research has been carried out over the last 20 years with a view to improving benzothiazine-based drugs and to avoid the adverse effects [6–7].

**Figure 1:**
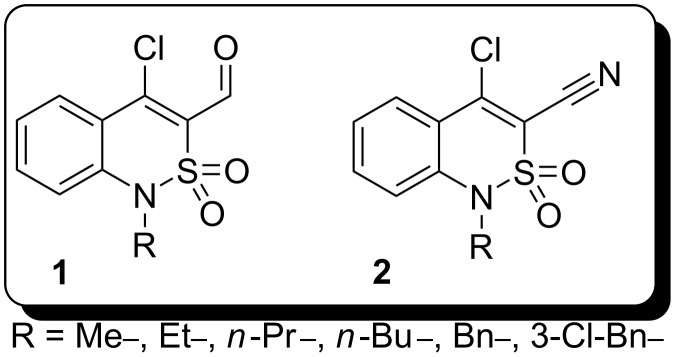
Benzo[*e*][2,1]thiazine-4-chloro-3-carbaldehydes **1** and benzo[*e*][2,1]thiazine-4-chloro-3-carbonitriles **2**.

In previous work [8–9] we reported the synthesis of some novel benzothiazines, in particular benzo[*e*][2,1]thiazine derivatives, the β-chloroaldehydes **1** and β-chloronitriles **2** (Figure 1); their chemistry was shown to be quite versatile. Compounds **1** and **2** both contain 1,3-dielectrophilic fragments, which are C-4 carbon atoms of the benzothiazine ring and carbonyl group or nitrile function, respectively. Thus, further investigation of aldehydes **1** and nitriles **2** should provide new benzo[*e*][2,1]thiazine derivatives, which can be used as intermediates for the synthesis of more elaborate compounds with potential biological activity.

The current article describes the chemical behaviour of chloroaldehydes **1a,b** and chloronitriles **2a,b** (Table 1) in oxidation and reduction reactions.

**Table 1:** Chloroaldehydes **1a,b** and chloronitriles **2a,b**.

Label		R

**1a**	**2a**	–Me
**1b**	**2b**	–Et

## Results and Discussion

**Scheme 1:**
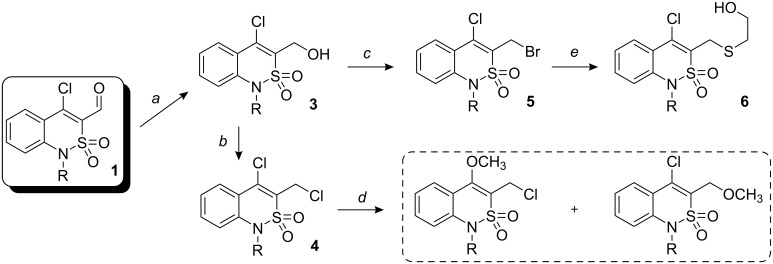
*a*: NaBH_4_ (2.5 equiv), MeOH, 2 h, room temp.; *b*: SOCl_2_ (4 equiv), benzene, 3 h, 0 °C to room temp.; *c*: HBr (50%), 5 h, reflux; *d*: NaOMe/MeOH, 2 h, reflux; *e*: HS–(CH_2_)_2_–OH (1.5 equiv), K_2_CO_3_, 1,4-dioxane, TEA, 2 h, reflux.

Chloroaldehydes **1a,b** are readily reduced under mild conditions by sodium borohydride to yield the alcohols **3a,b**. Treatment of compounds **3a,b** with thionyl chloride in dry benzene results in the formation of dichloro derivatives **4a,b**, whilst the 3-bromomethyl derivatives **5a,b** are obtained by refluxing **3a,b** in concentrated hydrobromic acid. Nucleophilic substitution of the chlorine atoms in compounds **4** shows similar behaviour. Thus, treatment of the dichloro derivatives **4a,b** with sodium methoxide gives a mixture of substitution products in a 2:1 isomer ratio with side-chain substitution predominating. The bromine atom in compounds **5** is much more reactive than the chlorines in **4**. Thus, when **5a,b** were heated with 2-mercaptoethanol and triethylamine in dioxane, compounds **6a,b** were obtained as the sole products (Scheme 1).

**Scheme 2:**
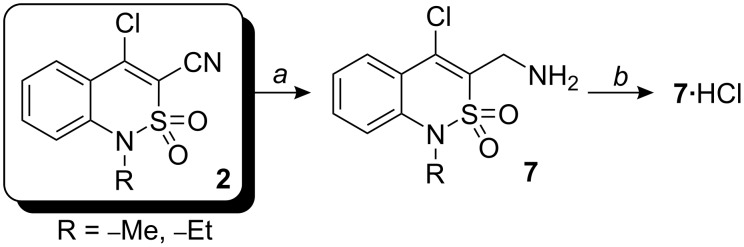
*a*: LiAlH_4_ (4 equiv), Et_2_O (THF), 3 h, 0 °C to room temp.; *b*: HCl saturated in 1,4-dioxane.

Amines **7a,b** were synthesized by reduction of chloronitriles **2a,b** with lithium aluminium hydride in dry diethyl ether or THF. Compounds **7** are very unstable. Presumably, intermolecular arylation of the active amino group occurs rapidly. Consequently, amines **7a,b** were isolated as their hydrochlorides (Scheme 2).

**Scheme 3:**
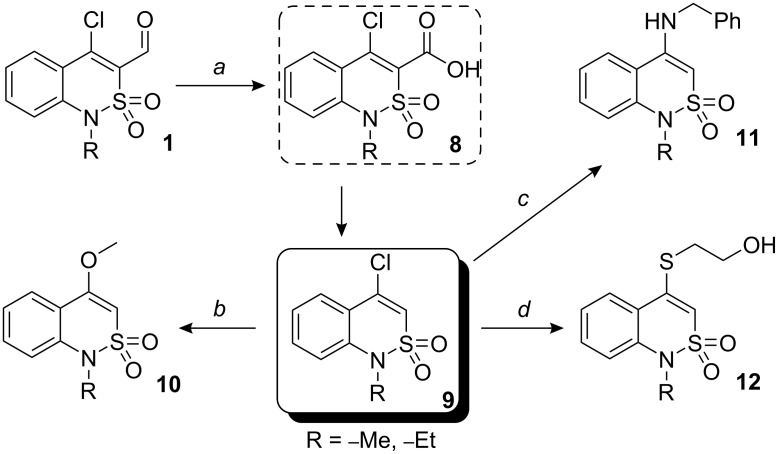
*a*: AgNO_3_ (1.5 equiv), NaOH, H_2_O/CH_2_Cl_2_, 3 h, room temp.; *b*: NaOMe/MeOH, 1 h, reflux; *c*: PhCH_2_NH_2_ (2 equiv), i-PrOH, 2 h, reflux; *d*: HS–(CH_2_)_2_–OH (1.5 equiv), K_2_CO_3_, dioxane, TEA, 1 h, reflux.

Despite our expectations, aldehydes **1** appeared to be inert to the most common oxidizing agents. Thus, oxidants such as sodium dichromate and potassium permanganate did not transform **1** into the corresponding carboxylic acids. Attempted oxidation of compounds **1** with hydrogen peroxide, peroxyacetic acid and *m*-chloroperoxybenzoic acid (*m*CPBA) was also unsuccessful. The desired oxidation reaction occurs with silver(I) oxide, prepared *in situ* from silver nitrate. Aldehydes **1a,b** are oxidized under mild conditions; however, the resulting carboxylic acids **8** could not be isolated, since even at low temperatures (up to 3 °C) and with immediate adjustment of the pH (between pH = 10 and pH = 3) instantaneous decarboxylation was observed, and 4-chlorobenzo[*e*][2,1]thiazines **9a,b** were obtained in quantitative yield. The chlorine atom in compounds **9a,b** is readily substituted by O-, N- and S-nucleophiles. Reaction of **9a,b** with sodium methoxide, benzylamine, and 2-mercaptoethanol gave the corresponding 4-methoxybenzothiazines **10a,b**, *N*-benzylamines **11a,b** and sulfanylethanol derivatives **12a,b**, respectively (Scheme 3).

## Conclusion

In summary, we describe the preparation of novel benzo[*e*][2,1]thiazine derivatives capable of further modification. In particular β-hydroxymethylchlorides **3a,b**, amines **7a,b** and 4-chlorobenzo[*e*][2,1]thiazines **9a,b** were obtained in high yields (95–99%) via convenient protocols involving oxidation and reduction reactions of compounds **1** and **2**. Biological testing of the synthesized compounds is currently in progress. The structures of all the described products (Table 2) were established from their NMR spectra, elemental analyses and mass spectra.

**Table 2:** Representative compounds prepared via Schemes 1, 2 and 3.

Product label	Yield (%)	
	
	**a** (R = –Me)	**b** (R = –Et)

**3**	93	95
**4**	95	98
**5**	71	76
**6**	55	57
**7**	67	68
**9**	99	99
**10**	85	87
**11**	60	63
**12**	32	34

## Experimental

### General procedure for the synthesis of compounds **3**

To a magnetically stirred suspension of **1a,b** (1 g, 4 mmol) in dry methanol (5 mL) sodium borohydride (0.64 g, 16 mmol) was added in small portions at room temperature. The solution was stirred for 2 hours and concentrated *in vacuo*. Dilute hydrochloric acid (2N HCl, 5 mL) was added, the solid product filtered off and washed with water to give pure compound **3a,b** (93–95%).

### General procedure for the synthesis of compounds **4**

To a magnetically stirred suspension of **3a,b** (1 g, 3.6 mmol) in dry benzene (6 mL) thionyl chloride (1.5 mL) was added dropwise at room temperature. The solution was stirred for 3 hours and concentrated *in vacuo*. Water was added and the solid product filtered off. Pure **4a,b** was obtained by crystallization from hexane (95–98%).

### General procedure for the synthesis of compounds **5**

The mixture of **3a,b** (1 g, 3.6 mmol) and concentrated hydrobromic acid (3 mL) was refluxed for 5 hours. The reaction mixture was cooled to 0 °C, the solids filtered off and washed with water to give pure compound **5a,b** (71–76%).

### General procedure for the synthesis of compounds **6**

The mixture of compound **5a,b** (1 g, 2.6 mmol), mercaptoethanol (0.5 mL), potassium carbonate (0.5 g) and triethylamine (1.5 mL) in 1,4-dioxane (5 mL) was refluxed for 2 hours, then concentrated *in vacuo* to afford the crude product as a brown oil. The pure product **6a,b** was obtained by column chromatography (CHCl_3_/CH_3_OH 9:1, 55–57%).

### General procedure for the synthesis of compounds **7**

To a magnetically stirred suspension of **2a,b** (1 g, 4 mmol) in dry ether (3 mL) or dry THF (3 mL) lithium aluminium hydride (0.7 g, 16 mmol) was added in small portions at 0 °C. The solution was stirred for 3 hours and the temperature gradually raised to 25 °C. The solvent was evaporated and water added to the residue. The product was extracted with CH_2_Cl_2_ (3 × 5 mL), concentrated *in vacuo* and treated with dry 1,4-dioxane saturated with HCl to afford the pure crystalline product **7a,b** (67–68%).

### General procedure for the synthesis of compounds **9**

To a magnetically stirred aqueous solution of silver nitrate (1 g, 6 mmol) sodium hydroxide (0.3 g, 7.5 mmol) was added in small portions at room temperature. To the resulting suspension of Ag_2_O a solution of **1a,b** (1 g, 4 mmol) in CH_2_Cl_2_ (3 mL) was added dropwise and the resulting mixture stirred for 3 hours. The organic layer was separated and concentrated *in vacuo* to give the pure solid product **9a,b** (99%).

### General procedure for the synthesis of compounds **10**

The mixture of **9a,b** (1 g, 4 mmol) and MeONa (0.5 g, 8.5 mmol) in dry methanol (5 mL) was refluxed for 1 hour. The solvent was evaporated and the solid product washed with i-PrOH to give pure compound **10a,b** (85–87%).

### General procedure for the synthesis of compounds **11**

The mixture of **9a,b** (1 g, 4 mmol) and benzylamine (0.5 g, 5 mmol) in i-PrOH (5 mL) was refluxed for 2 hours. The solvent was evaporated, and the solid product washed with i-PrOH and water. The pure product **11a,b** was obtained by crystallization from toluene (60–63%).

### General procedure for the synthesis of compounds **12**

The mixture of **9a,b** (1 g, 4 mmol), mercaptoethanol (0.5 mL), potassium carbonate (0.5 g) and triethylamine (1.5 mL) in 1,4-dioxane (7 mL) was refluxed for 1 hour. Inorganic material was removed by filtration and the resulting solution concentrated *in vacuo*. The oily product was purified by column chromatography on silica gel with chloroform-methanol (8:2) as eluent to give the pure product **12a,b** (32–34%).

## Supporting Information

Supporting information contains melting points, elemental analyses, ^1^H-, ^13^C-NMR and mass spectra data for all new compounds (**3**–**12**).

File 1 Spectroscopic data for: On the functionalization of benzo[*e*][2,1]thiazine.
