# Supplementary material for: On the functionalization of benzo[e][2,1]thiazine
Source: Beilstein J Org Chem. 2009 Sep 2;5:42. doi: 10.3762/bjoc.5.42 (PMC2779739; doi:10.3762/bjoc.5.42)
Supplement: File 1 — Spectroscopic data for: On the functionalization of benzo[e][2,1]thiazine. [file Beilstein_J_Org_Chem-05-42-s001.doc]

# Supporting Information

# for:

# On the functionalization of benzo[*e*][2,1]thiazine

Kirill Popov*, Tatyana Volovnenko and Julian Volovenko

Address: Organic Chemistry Department, Kiev State University, Kiev, 01033, Ukraine

Email: Kirill Popov* - popovkirill@bk.ru

* Corresponding author

The ^1^H- and ^13^C-NMR spectra were measured on a Varian Unity Plus 400 spectrometer using DMSO-*d_6_* as solvent. All chemical shifts are reported in ppm relative to TMS. Mass spectra were registred on Agilent 1100 LCMSD SL instrument. Column chromatography was conducted on Kieselgel Merck 60 (230 – 400 mesh) (silica gel). Starting materials were obtained from Merck and used without further purification. Dry solvents were prepared according to the standard methods.

**Spectroscopic and analytical data**

(4-chloro-1-methyl-1*H*-2,1-benzothiazin-2,2-dioxide-3-yl)methanol (**3a**)

m.p.: 211-212 ˚C; ^1^H-NMR (400 MHz, DMSO-D_6_) δ = 3.88 (s, 3H), 4.63 (s, 2H), 5.59 (s, 1H), 7.34 (t, *J = 8 Hz*, 1H), 7.49 (d, *J = 8.2 Hz*, 1H, 7.61 (t, *J = 8 Hz*, 1H), 7.94 (d, *J = 8.2 Hz*, 1H); ^13^C-NMR (100 MHz, DMSO-D6) δ = 39.23, 57.75, 118.57, 122.61, 124.70, 128.13, 132.19, 136.58, 137.84, 139.05; HRMS (EI-TOF): m/z [M^+^] Calcd. 259.0070; Found 259.0064; Anal. calcd. C, 46.25; H, 3.88; N, 5.39; found C, 46.27; H, 3.91; N, 5.41.

(4-chloro-1-ethyl-1*H*-2,1-benzothiazin-2,2-dioxide-3-yl)methanol (**3b**)

m.p.: 192-193 ˚C; ^1^H-NMR (400 MHz, DMSO-D_6_) δ = 1.28 (t, *J = 6.8 Hz*, 3H), 4.04 (q, *J = 6.8 Hz*, 2H), 4.63 (s, 2H), 5.59 (s, 1H), 7.34 (t, *J = 8 Hz*, 1H), 7.49 (d, *J = 8.2 Hz*, 1H, 7.61 (t, *J = 8 Hz*, 1H), 7.94 (d, *J = 8.2 Hz*, 1H); ^13^C-NMR (100 MHz, DMSO-D6) δ = 14.45, 43.12, 57.78, 118.56, 122.62, 124.71, 128.13, 132.18, 136.55, 137.80, 139.03; HRMS (EI-TOF): m/z [M^+^] Calcd. 273.0226; Found 273.0221; Anal. calcd. C, 48.26; H, 4.42; N, 5.12; found C, 48.29; H, 4.44; N, 5.15.

4-chloro-3-(chloromethyl)-1-methyl-1*H*-2,1-benzothiazin-2,2-dioxide (**4a**)

m.p.: 239-240 ˚C; ^1^H-NMR (400 MHz, DMSO-D_6_) δ = 3.93 (s, 3H), 4.79 (s, 2H), 7.40 (t, *J = 7.6 Hz*, 1H), 7.57 (d, *J = 7.8 Hz*, 1H, 7.63 (t, *J = 7.6 Hz*, 1H), 7.97 (d, *J = 7.8 Hz*, 1H); ^13^C-NMR (100 MHz, DMSO-D6) δ = 38.03, 50.74, 118.45, 122.67, 124.65, 128.12, 132.21, 136.57, 137.82, 139.04; HRMS (EI-TOF): m/z [M^+^] Calcd. 276.9731; Found 276.9726; Anal. calcd. C, 43.18; H, 3.26; N, 5.04; found C, 43.20; H, 3.27; N, 5.07.

4-chloro-3-(chloromethyl)-1-ethyl-1*H*-2,1-benzothiazin-2,2-dioxide (**4b**)

m.p.: 223-224 ˚C; ^1^H-NMR (400 MHz, DMSO-D_6_) δ = 1.29 (t, *J = 6.8 Hz*, 3H), 4.07 (q, *J = 6.8 Hz*, 2H), 4.79 (s, 2H), 7.40 (t, *J = 7.6 Hz*, 1H), 7.57 (d, *J = 7.8 Hz*, 1H, 7.63 (t, *J = 7.6 Hz*, 1H), 7.97 (d, *J = 7.8 Hz*, 1H); ^13^C-NMR (100 MHz, DMSO-D6) δ = 14.48, 42.13, 50.71, 118.46, 122.67, 124.67, 128.17, 132.24, 136.59, 137.87, 139.02; HRMS (EI-TOF): m/z [M^+^] Calcd. 290.9888; Found 290.9883; Anal. calcd. C, 45.22; H, 3.79; N, 4.79; found C, 45.24; H, 3.82; N, 4.81.

3-(bromomethyl)-4-chloro-1-methyl-1*H*-2,1-benzothiazin-2,2-dioxide (**5a**)

m.p.: 245-246 ˚C; ^1^H-NMR (400 MHz, DMSO-D_6_) δ = 4.01 (s, 3H), 4.66 (s, 2H), 7.39 (t, *J = 8 Hz*, 1H), 7.56 (d, *J = 8.2 Hz*, 1H), 7.66 (t, *J = 8 Hz*, 1H), 7.96 (d, *J = 7.8 Hz*, 1H); ^13^C-NMR (100 MHz, DMSO-D6) δ = 37.92, 43.12, 118.47, 122.57, 124.69, 128.04, 132.10, 136.51, 138.01, 138.92; HRMS (EI-TOF): m/z [M^+^] Calcd. 320.9226; Found 320.9221; Anal. calcd. C, 37.23; H, 2.81; N, 4.34; found C, 37.27; H, 2.84; N, 4.33.

3-(bromomethyl)-4-chloro-1-ethyl-1*H*-2,1-benzothiazin-2,2-dioxide (**5b**)

m.p.: 231-232 ˚C; ^1^H-NMR (400 MHz, DMSO-D_6_) δ = 1.27 (t, *J = 6.8 Hz*, 3H), 4.06 (q, *J = 6.8 Hz*, 2H), 4.66 (s, 2H), 7.39 (t, *J = 8 Hz*, 1H), 7.56 (d, *J = 8.2 Hz*, 1H), 7.66 (t, *J = 8 Hz*, 1H), 7.96 (d, *J = 7.8 Hz*, 1H); ^13^C-NMR (100 MHz, DMSO-D6) δ = 14.67, 43.02, 44.08, 118.50, 122.64, 124.75, 128.14, 132.19, 136.57, 137.87, 139.03; HRMS (EI-TOF): m/z [M^+^] Calcd. 334.9382; Found 334.9387; Anal. calcd. C, 39.25; H, 3.29; N, 4.16; found C, 39.27; H, 3.32; N, 4.19.

2-{[(4-chloro-1-methyl-1*H*-2,1-benzothiazin-2,2-dioxide-3-yl)methyl]sulfanyl}-1-ethanol (**6a**)

Oil; ^1^H-NMR (400 MHz, DMSO-D_6_) δ = 2.84 (t, *J = 7 Hz*, 2H), 3.71 (t, *J = 6.8 Hz*, 2H), 3.89 (s, 2H), 3.95 (s, 3H), 5.00-5.40 (br.s, 1H), 7.19-7.24 (m, 2H), 7.49 (t, *J = 7.6 Hz*, 1H), 7.92 (d, *J = 7.8 Hz*, 1H); ^13^C-NMR (100 MHz, DMSO-D6) δ = 36.12, 38.33, 39.22, 58.10, 118.40, 122.51, 124.69, 128.38, 132.50, 136.67, 137.93, 139.15; HRMS (EI-TOF): m/z [M^+^] Calcd. 319.0104; Found 319.0100; Anal. calcd. C, 45.06; H, 4.41; N, 4.38; found C, 45.08; H, 4.44; N, 4.41.

2-{[(4-chloro-1-ethyl-1*H*-2,1-benzothiazin-2,2-dioxide-3-yl)methyl]sulfanyl}-1-ethanol (**6b**)

Oil; ^1^H-NMR (400 MHz, DMSO-D_6_) δ = 1.40 (t, *J = 6.8 Hz*, 3H), 2.84 (t, *J = 7 Hz*, 2H), 3.71 (t, *J = 6.8 Hz*, 2H), 3.89 (s, 2H), 4.03 (q, *J = 7 Hz*, 2H), 5.00-5.40 (br.s, 1H), 7.19-7.24 (m, 2H), 7.49 (t, *J = 7.6 Hz*, 1H), 7.92 (d, *J = 7.8 Hz*, 1H); ^13^C-NMR (100 MHz, DMSO-D6) δ = 14.61, 36.12, 38.33, 41.34, 58.08, 118.42, 122.53, 124.71, 128.42, 132.52, 136.70, 137.95, 139.16; HRMS (EI-TOF): m/z [M^+^] Calcd. 333.0260; Found 333.0264; Anal. calcd. C, 46.77; H, 4.83; N, 4.20; found C, 46.79; H, 4.86; N, 4.21.

(4-chloro-1-methyl-1*H*-2,1-benzothiazin-2,2-dioxide-3-yl)methanamine hydrochloride (**7a**)

m.p.: decomp. on heating; ^1^H-NMR (400 MHz, DMSO-D_6_) δ = 3.94 (s, 3H), 6.05 (br. s, 2H), 7.33 (t, *J = 8 Hz*, 1H), 7.50 (d, *J = 8.2 Hz*, 1H), 7.64 (t, *J = 7.8 Hz*, 1H), 7.90 (d, *J = 8 Hz*, 1H); ^13^C-NMR (100 MHz, DMSO-D6) δ = 55.06, 117.92, 122.01, 124.25, 127.85, 132.12, 136.22, 137.34, 138.88; HRMS (EI-TOF): m/z [M^+^] Calcd. 293.9997; Found 293.9992; Anal. calcd. C, 40.69; H, 4.10; N, 9.49; found C, 40.72; H, 4.11; N, 9.52.

(4-chloro-1-ethyl-1*H*-2,1-benzothiazin-2,2-dioxide-3-yl)methanamine hydrochloride (**7b**)

m.p.: decomp. on heating; ^1^H-NMR (400 MHz, DMSO-D_6_) δ = 1.30 (t, *J = 6.8 Hz*, 3H), 4.06 (q, *J = 6.8 Hz*, 2H), 6.05 (br. s, 2H), 7.33 (t, *J = 8 Hz*, 1H), 7.50 (d, *J = 8.2 Hz*, 1H), 7.64 (t, *J = 7.8 Hz*, 1H), 7.90 (d, *J = 8 Hz*, 1H); ^13^C-NMR (100 MHz, DMSO-D6) δ = 14.59, 42.01, 55.08, 117.90, 121.99, 124.21, 127.89, 131.99, 136.05, 137.35, 138.85; HRMS (EI-TOF): m/z [M^+^] Calcd. 308.0153; Found 308.0158; Anal. calcd. C, 42.73; H, 4.56; N, 9.06; found C, 42.75; H, 4.58; N, 9.09.

4-chloro-1-methyl-1*H*-2,1-benzothiazin-2,2-dioxide (**9a**)

m.p.: 76-77 ˚C; ^1^H-NMR (400 MHz, DMSO-D_6_) δ = 3.89 (s, 3H), 7.33 (t, *J = 8 Hz*, 1H), 7.50 (d, *J = 8.2 Hz*, 1H), 7.64 (t, *J = 7.8 Hz*, 1H), 7.68 (s, 1H), 7.90 (d, *J = 8 Hz*, 1H); ^13^C-NMR (100 MHz, DMSO-D6) δ = 37.15, 117.91, 120.04, 122.88, 123.33, 127.36, 132.17, 138.02, 138.92; HRMS (EI-TOF): m/z [M^+^] Calcd. 228.9964; Found 228.9961; Anal. calcd. C, 47.06; H, 3.51; N, 6.10; found C, 47.08; H, 3.54; N, 6.12.

4-chloro-1-ethyl-1*H*-2,1-benzothiazin-2,2-dioxide (**9b**)

m.p.: 62-63 ˚C; ^1^H-NMR (400 MHz, DMSO-D_6_) δ = 1.30 (t, *J = 6.8 Hz*, 3H), 4.06 (q, *J = 6.8 Hz*, 2H), 7.33 (t, *J = 8 Hz*, 1H), 7.50 (d, *J = 8.2 Hz*, 1H), 7.64 (t, *J = 7.8 Hz*, 1H), 7.68 (s, 1H), 7.90 (d, *J = 8 Hz*, 1H); ^13^C-NMR (100 MHz, DMSO-D6) δ = 14.66, 41.34, 118.34, 120.74, 123.18, 123.53, 127.77, 132.88, 138.42, 139.04; HRMS (EI-TOF): m/z [M^+^] Calcd. 243.0121; Found 243.0126; Anal. calcd. C, 49.28; H, 4.14; N, 5.75; found C, 49.31; H, 4.18; N, 5.73.

1-methyl-4-methoxy-1*H*-2,1-benzothiazin-2,2-dioxide (**10a**)

m.p.: 120-121 ˚C; ^1^H-NMR (400 MHz, DMSO-D_6_) δ = 3.87 (s, 3H), 3.96 (s, 3H), 6.06 (s, 1H), 7.18 (t, *J = 8 Hz*, 1H), 7.34 (d, *J = 8.2 Hz*, 1H), 7.54 (t, *J = 7.8 Hz*, 1H), 7.81 (d, *J = 8 Hz*, 1H); ^13^C-NMR (100 MHz, DMSO-D6) δ = 39.03, 57.03, 101.41, 117.43, 118.99, 123.07, 126.01, 132.73, 138.92, 159.12; HRMS (EI-TOF): m/z [M^+^] Calcd. 225.0460; Found 225.0455; Anal. calcd. C, 53.32; H, 4.92; N, 6.22; found C, 53.35; H, 4.90; N, 6.24.

1-ethyl-4-methoxy-1*H*-2,1-benzothiazin-2,2-dioxide (**10b**)

m.p.: 105-106 ˚C; ^1^H-NMR (400 MHz, DMSO-D_6_) δ = 1.27 (t, *J = 6.8 Hz*, 3H), 3.87 (s, 3H), 4.02 (q, *J = 6.8 Hz*, 2H), 6.06 (s, 1H), 7.18 (t, *J = 8 Hz*, 1H), 7.34 (d, *J = 8.2 Hz*, 1H), 7.54 (t, *J = 7.8 Hz*, 1H), 7.81 (d, *J = 8 Hz*, 1H); ^13^C-NMR (100 MHz, DMSO-D6) δ = 14.36, 40.98, 57.01, 100.34, 117.46, 118.91, 122.42, 125.22, 132.05, 138.29, 158.86; HRMS (EI-TOF): m/z [M^+^] Calcd. 239.0616; Found 239.0611; Anal. calcd. C, 55.21; H, 5.48; N, 5.85; found C, 55.24; H, 5.51; N, 5.88.

*N*-benzyl-1-methyl-1*H*-2,1-benzothiazin-4-amin-2,2-dioxide (**11a**)

m.p.: 221-222 ˚C; ^1^H-NMR (400 MHz, DMSO-D_6_) δ = 3.89 (s, 3H), 4.76 (s, 2H), 6.20 (s, 1H), 7.03 (br.s, 1H), 7.17-7.26 (m, 5H), 7.42-7.51 (m, 2H), 7.68 (t, *J = 7.8 Hz*, 1H), 7.93 (d, *J = 8 Hz*, 1H); ^13^C-NMR (100 MHz, DMSO-D6) δ = 37.15, 46.47, 117.90, 120.07, 122.89, 123.37, 126.72, 127.03, 127.08, 127.56, 128.42, 128.55, 132.07, 138.09, 139.02, 143.38; HRMS (EI-TOF): m/z [M^+^] Calcd. 300.0932; Found 300.0928; Anal. calcd. C, 63.98; H, 5.37; N, 9.33; found C, 64.01; H, 5.39; N, 9.35.

*N*-benzyl-1-ethyl-1*H*-2,1-benzothiazin-4-amin-2,2-dioxide (**11b**)

m.p.: 204-205 ˚C; ^1^H-NMR (400 MHz, DMSO-D_6_) δ = 1.26 (t, *J = 6.8 Hz*, 3H), 4.02 (q, *J = 6.8 Hz*, 2H), 4.76 (s, 2H), 6.20 (s, 1H), 7.03 (br.s, 1H), 7.17-7.26 (m, 5H), 7.42-7.51 (m, 2H), 7.68 (t, *J = 7.8 Hz*, 1H), 7.93 (d, *J = 8 Hz*, 1H); ^13^C-NMR (100 MHz, DMSO-D6) δ = 14.68, 41.31, 46.41, 118.14, 120.64, 123.19, 123.93, 126.70, 127.04, 127.09, 127.73, 128.46, 128.53, 132.78, 138.43, 139.07, 143.35; HRMS (EI-TOF): m/z [M^+^] Calcd. 314.1089; Found 314.1083; Anal. calcd. C, 64.94; H, 5.77; N, 8.91; found C, 64.97; H, 5.80; N, 8.94.

2-[(1-methyl-1*H*-2,1-benzothiazin-2,2-dioxide-4-yl)sulfanyl]-1-ethanol (**12a**)

Oil; ^1^H-NMR (400 MHz, DMSO-D_6_) δ = 3.12 (t, *J = 6.8 Hz*, 2H), 3.67 (t, *J = 7.2 Hz*, 2H), 3.92 (s, 3H), 6.95 (s, 1H), 7.23 (t, *J = 8 Hz*, 1H), 7.40 (d, *J = 8.2 Hz*, 1H), 7.54 (t, *J = 7.8 Hz*, 1H), 7.82 (d, *J = 7.8 Hz*, 1H), 8.15 (br. s, 1H); ^13^C-NMR (100 MHz, DMSO-D6) δ = 34.60, 39.27, 41.40, 116.18, 119.02, 122.05, 123.28, 126.53, 131.81, 138.30, 146.29; HRMS (EI-TOF): m/z [M^+^] Calcd. 271.0337; Found 271.0333; Anal. calcd. C, 48.69; H, 4.83; N, 5.16; found C, 48.72; H, 4.85; N, 5.19.

2-[(1-ethyl-1*H*-2,1-benzothiazin-2,2-dioxide-4-yl)sulfanyl]-1-ethanol (**12b**)

Oil; ^1^H-NMR (400 MHz, DMSO-D_6_) δ = 1.26 (t, *J = 6.8 Hz*, 3H), 3.12 (t, *J = 6.8 Hz*, 2H), 3.67 (t, *J = 7.2 Hz*, 2H), 3.99 (q, *J = 7.2 Hz*, 2H), 6.95 (s, 1H), 7.23 (t, *J = 8 Hz*, 1H), 7.40 (d, *J = 8.2 Hz*, 1H), 7.54 (t, *J = 7.8 Hz*, 1H), 7.82 (d, *J = 7.8 Hz*, 1H), 8.15 (br. s, 1H); ^13^C-NMR (100 MHz, DMSO-D6) δ = 14.54, 34.60, 41.42, 59.09, 116.11, 118.78, 122.03, 123.23, 126.49, 131.80, 138.35, 146.19; HRMS (EI-TOF): m/z [M^+^] Calcd. 285.0493; Found 285.0497; Anal. calcd. C, 50.50; H, 5.30; N, 4.91; found C, 50.52; H, 5.33; N, 4.92.
